# Reframing diabetes mellitus care in South Africa through lived experience, peer support and advocacy: Lessons from the 2025 Diabetes Summit

**DOI:** 10.4102/phcfm.v18i2.5578

**Published:** 2026-08-17

**Authors:** Melanie A. Pienaar, Alexandra van Essche, Sandhya Singh, Patrick Ngassa Piotie, Kibachio J. Mwangi

**Affiliations:** 1 School of Nursing, Faculty of Health Sciences, University of the Free State, Bloemfontein, South Africa; 2 Diabetes Alliance, Pretoria, South Africa; 3 School of Health Systems and Public Health, Faculty of Health Sciences, University of Pretoria, Pretoria, South Africa; 4 Department of Public Health, Medical School, Nelson Mandela University, Gqeberha, South Africa; 5 World Health Organization, Pretoria, South Africa

## Introduction

The prevalence of diabetes mellitus (DM) in South Africa is estimated at 7.2% among adults aged 20–79 years, equating to approximately 2.3 million individuals living with the condition. Notably, up to 70% of cases in South Africa remain undiagnosed.^1^ Given the substantial burden of DM and the high proportion of undiagnosed cases in South Africa, the meaningful inclusion of individuals with a lived experience of DM is essential to inform responsive, patient-centred approaches and to drive transformation within healthcare systems. The World Health Organization emphasises the role of individuals with lived experience who have firsthand knowledge of a social issue, health condition or disability, in forming patient-centred approaches and transformation within healthcare systems.^2^

The Diabetes Alliance is a non-profit organisation with a mission to unite stakeholders within South Africa and globally to reduce the burden of DM through advocacy, education, community engagement and collaborative partnerships. On 11–13 November 2025, the Diabetes Alliance convened the third Diabetes Summit at the Radisson Hotel & Convention Centre in Johannesburg. The theme of the Summit was ‘Innovate for Impact: Transforming the Future of Diabetes in South Africa’, which was explored through various panel discussions and tracks. In the words of the Chairperson of the Diabetes Alliance, Dr Patrick Ngassa Piotie: ‘Innovation is not only about technology, but also about transforming lives. It is about ensuring that every person with DM, regardless of where they live or what they earn, has access to quality care, education, and hope’.

This report will focus on the two lived-experience tracks presented during the Diabetes Summit.

## Diabetes Summit lived experience track: South African Depression and Anxiety Group

The South African Depression and Anxiety Group (SADAG) facilitated the first lived experience track on the first day of the Diabetes Summit. The group has a 30-year track record in providing support to South Africans struggling with their mental health and has expanded its services to support people living with non-communicable diseases (NCDs), including DM.

The following points were raised.

### Vulnerability to mental illness

As confirmed by presenters and delegates, individuals living with chronic diseases such as DM are more vulnerable to experiencing mental health challenges because of the lifelong and demanding nature of the condition, and the associated stigma and fear.^3,4^ Living with DM requires continuous and ongoing commitment because it involves a constant focus on blood glucose testing and/or monitoring, adhering to medication regimes, eating a healthy diet and remaining physically active.

Ensuing discussion strongly supported that DM stigma is a reality and refers to judgement, blame, prejudice^5^ and social disapproval^4^ against individuals living with DM. Stigma may occur in various work, home, health, educational and interpersonal settings. Individuals living with DM who are overweight or obese are specifically vulnerable to stigma.

Stigma is based on the misconception that the diagnosis of DM occurred because of unhealthy lifestyle decisions.^6^ The role of broader social and economic determinants, such as poverty, food insecurity and unemployment, that shape lifestyle choices is often ignored. Studies also suggest that individuals living with DM often face self-stigma in the form of self-blame, shame and guilt.^5^ The International Diabetes Federation identified DM stigma as a critical challenge that must be addressed.^7^ An international review of DM stigma and clinical outcomes demonstrated high prevalence rates of stigma in adults with type one and type two DM (78% and 70%, respectively). Furthermore, DM stigma has a devastating emotional impact that is associated with poor self-management behaviours, poor glycaemic control evidenced by raised glycated hemoglobin (HbA1C) levels and increased prevalence of complications.^3,8^ Essentially, DM stigma may be a critical barrier to care and self-management and needs to be recognised and addressed in the same way that human immunodeficiency virus (HIV) and acquired immunodeficiency syndrome (AIDS) stigma was and is being addressed.

Delegates agreed that individuals living with DM experience constant fear and anxiety over the unpredictable nature of their condition: low and high blood glucose levels, complications, worrying about being sick and not being able to go to work, money for medication, the future, their family and not being able to do everything that other people do. Individuals living with DM express emotional stress^9^ and DM burnout – they feel physically, emotionally and mentally exhausted.^10^ This has an impact on their ability to self-manage their DM.

### Value of peer support

Presenters and delegates highlighted that peer support, therefore, becomes a powerful tool^11^ to navigate the psychological, informational and emotional turmoil experienced by individuals with DM.^12^ Peer support can be rendered in various ways by individuals who have lived knowledge, experience or similar circumstances, such as family members, caregivers, healthcare providers, teachers, support groups, diabetes nurse educators and community health workers.^12,13^ The value of peer support lies in sharing experiences, practical coping strategies, improving knowledge and self-management skills, and building a supportive community.^11^ Peer support may be associated with improved self-management behaviour, self-efficacy and clinical outcomes.^14,15^

## Diabetes Summit lived experience track: Healthy Living Alliance

The second workshop focused on Advocacy for Prevention and Care – Building Momentum for Policy Change. The workshop was hosted by Healthy Living Alliance (HEALA). Healthy Living Alliance is a South African coalition of civil society organisations that work together with the government and other stakeholders to promote wellness and improve public health by advocating for equitable access to affordable, nutritious food and healthier food environments for all (including a focus on addressing obesity in the country).

This session explored the impact of an unaffordable and unhealthy food environment on the onset and perpetuation of NCDs. Globally and in South Africa, obesity is a major risk factor for NCDs, especially DM and is increasingly framed as a chronic, complex and recurring disease.^16^ However, despite this recognition, it remains inadequately prioritised within policy and health systems responses. The publication of South Africa’s first national clinical practice guideline for obesity management in 2025 represents a critical step forward,^17^ but coordinated, ‘whole of government’ and ‘whole of society’ action is still required to address obesity holistically.

### Food justice and dietary choices

Healthy Living Alliance strongly promotes food justice, recognising that what people eat profoundly affects their health and wellbeing as dietary choices are often shaped by broader social, commercial and economic determinants of health. In this regard, the powerful food industry plays a dominant role in perpetuating poor health and escalating NCDs to maintain profit margins. Ultra-processed products are high in trans fats, sugar and/or salt, and other additives, which are easily accessible, especially to poorer communities, and may seem more affordable. Large food companies play a dominant role in driving obesity – their products are convenient, seemingly affordable and widely accessible, yet they drive unhealthy diets and are major contributors to rising obesity, DM and other NCDs. When income is limited, people choose food that is affordable and filling, yet it is not nutritious.

## Systemic and service delivery challenges

Delegates agreed that in addition to the circumstances and context in which they live, individuals living with DM face significant challenges related to the health system and service delivery factors, directly impacting compliance to treatment, accessibility to treatment and quality of care.

Contrary to the stipulations of the Integrated Clinical Services Management Model,^18^ the arrangement and functioning of many health facilities continue to promote vertically based, fragmented care. Health system weaknesses such as long waiting times, shortage of skilled healthcare providers in DM care, frequent occurrence of shortage of DM drugs and consumables, poor monitoring of both DM and early signs of DM complications, coupled with limited patient–healthcare provider consultation time, compromise the quality of care rendered.

Literature shows that time limits during consultations with healthcare providers may influence the patient’s understanding of DM and hinder patient treatment compliance. Additionally, the unavailability of consumables and medication leads to poor medication adherence and glycaemic control, negatively impacting health outcomes while increasing the risk of developing complications.^19^

## The knowledge gap of healthcare providers related to diabetes

Limited DM-specific knowledge and expertise among healthcare providers may affect individuals’ self-management, leading to poor adherence and diabetes control. This highlights the urgency for enhanced training and capacity development among all levels of healthcare providers. While existing, the optimal utilisation of Standard Treatment Guidelines remains suboptimal, and the adoption of person- and patient-centred approaches for NCDs is not prioritised. These gaps appear to perpetuate a practice among healthcare workers (HCWs) to stigmatise DM and other NCDs as personal failures by patients that they must manage.

Based on the session proceedings, workable solutions going forward may include the following.

### Establish an inclusive and participatory approach

- Ensure people living with DM and obesity are fully included in the co-created design and delivery of care.
- Recognise cultural and contextual sensitivity of persons and patients in all strategies.
- Create a safe and open environment related to disclosure about DM. Mitigate stigma against individuals with NCDs, including DM.

### Promote food justice

- Institutionalise a ‘whole of government’ and ‘whole of society’ approach to food access, affordability and equity.
- Advocate for a greater understanding among stakeholders, particularly the government, to create enabling environments to address obesity.

### Strengthen health system response

- Promote awareness among patients of their rights to access the package of care, including mental health services.
- Overcome the practice among HCWs to ‘blame’ patients for poor health outcomes.
- Build capacity by training nurses, community health workers, peer educators and other healthcare providers.
- Strengthen routine monitoring of DM and other NCDs by healthcare providers and enhance the urgency of their responses to address problems.
- Recognise and enhance the importance of community-based services and peer support.

## Take-home message

The lived-experience tracks of the 2025 Diabetes Summit underscored the need to reframe DM care in South Africa through a more holistic, people-centred lens. By centring lived experience, strengthening peer support, and advancing advocacy, stakeholders highlighted pathways to more responsive and equitable care.

These discussions reinforced the importance of a whole-of-society approach, where communities, healthcare providers, civil society and policymakers work together to address the complex and interrelated drivers of DM – and what is required to timeously diagnose, treat and refer people living with DM.

Elevating the voices of people living with DM is not only a matter of inclusion – it is essential for designing effective interventions, shaping policy and ultimately improving health outcomes.
